# Downregulation of Signaling-active IGF-1 by Dipeptidyl Peptidase IV (DPP-IV)

**Published:** 2010-12

**Authors:** Ching-Ting Lin, Hsiang-Yun Tang, Yu-San Han, Hui-Ping Liu, Shiu-Feng Huang, Chia-Hui Chien, John Shyy, Jeng-Jian Chiu, Xin Chen

**Affiliations:** 1 *Division of Biotechnology and Pharmaceutical Research, National Health Research Institutes, Taiwan (ROC);*; 2 *School of Chinese Medicine, China Medical University, Taichung, Taiwan (ROC);*; 3 *Departments of Cardiovascular and Thoracic Surgery, Chang Gung Memorial Hospital, Tao-Yuan, Taiwan (ROC);*; 4 *Division of Molecular and Genomic Medicine, National Health Research Institutes, Taiwan (ROC);*; 5 *Division of Biomedical Sciences, University of California, Riverside, California, USA;*; 6 *Division of Biomedical Engineering, National Health Research Institutes, Miaoli County, Taiwan (ROC)*

**Keywords:** dipeptidyl peptidase IV, insulin-like growth factor-1, IGF binding protein 3, IGF signaling pathway

## Abstract

Functioning as an extracellular protease, dipeptidyl peptidase IV (DPP-IV) preferentially cleaves the peptide bond after the penultimate proline residue. We report here that DPP-IV cleaves the first two amino acids from insulin-like growth factor 1 (IGF-1), revealed by mass spectrometry. The kinetic parameters of the proteolytic cleavage indicate that this reaction is physiologically relevant. Interestingly, truncated IGF-1 is less potent than the full-length protein in activating the IGF-1R, but binds more readily to IGF-binding protein 3 (IGFBP3). Quantitative RT–PCR showed that the level of DPP-IV mRNA is dramatically lower in lung squamous cell carcinoma tissues than in adjacent nonneoplastic lung tissues. However, this reduction was not observed in lung adenocarcinoma tissues. Our study suggests a possible link between IGF-1 and DPP-IV in cancer development in a specific tumor niche. A DPP-IV-related pathway may be important in mitigating IGF-1 signaling. Consequently, a robust IGF signaling pathway may accelerate early carcinogenesis in environments lacking DPP-IV.

## INTRODUCTION

Dipeptidyl peptidase IV (DPP-IV) is a membrane-bound serine protease that preferentially cleaves the peptide bond after the penultimate proline residue (1). Its catalytic domain is located extracellularly, anchored to the membrane by a single transmembrane domain. DPP-IV dimerization is essential for its enzymatic activity (2–4). The substrates of DPP-IV are small oligopeptides, including hormones, chemokines, and neuropeptides (5–8). DPP-IV is ubiquitously expressed in various tissues. Interestingly, the enzymatic activity of DPP-IV is reduced in the sera of patients with melanomas or oral cancers (9, 10). Attenuated DPP-IV expression on the cell surface is also observed in malignant cells, such as melanomas and lung nonadenocarcinomas (10–15). In melanocytes, the elimination of DPP-IV occurs concomitantly with growth factor independence (14, 15). Re-expression of DPP-IV in these melanoma cells suppresses the malignant phenotype of the melanocytic cells (16). In human nonadenocarcinoma cancers, the enzymatic activity and expression of DPP-IV is dramatically reduced compared with those in normal bronchial and lung alveolar epithelia (11). Interestingly, the reexpression of DPP-IV in lung cancer cells inhibits tumor events, such as cell proliferation, anchorage-independent growth, migration, and tumorigenicity, in nude mice (17). The mechanisms of DPP-IV downregulation and tumor growth in these tissues are not yet clear.

Insulin-like growth factor 1 (IGF-1) is a powerful mitogen, stimulating cell proliferation (18, 19). A single polypeptide of 70 amino acids, IGF-1 binds to the IGF-1 receptor (IGF-1R) with high affinity (20, 21). The biological function of IGF-1 is mediated through IGF-1R tyrosine kinase signaling. IGF-1R phosphorylates several downstream adaptor proteins, thus activating distinct downstream signaling pathways, including the AKT, RAF, and MAPK pathways (22). The physiological concentration of IGF-1 is tightly regulated. The total concentration of IGF-1 in the blood is 10–40 nM (23–25). The IGF-1 concentration is controlled by IGF-binding proteins (IGFBPs), which bind free IGF-1 (23–25). Among these, IGFBP3 is the main IGF-1-sequestering protein, because of its abundance and high affinity for IGF-1 (21, 26). The remaining free IGF-1 constitutes less than 1% of the total IGF-1 concentration (23, 26). Given the tight binding between IGF-1 and IGFBPs (K_d_=0.06–0.22 nM) (21), the sequestering of IGF-1 by IGFBPs effectively reduces the amount of IGF-1 available to activate IGF-1R. In this way, IGFBPs negatively regulate the bioavailability of IGF-1.

In this study, IGF-1 was found to be proteolytically processed by the enzyme DPP-IV, resulting in a truncated form of IGF-1 (T-IGF1). Shortened by two amino acids, T-IGF1 is less potent in activating IGF-1R and binds more tightly to IGFBP3. Quantitative RT–PCR also showed that DPP-IV expression is significantly downregulated in human lung squamous cell carcinoma (SCC) tissues compared with that in adjacent nonneoplastic lung tissues. This study suggests a new and interesting link between DPP-IV and IGF-1 in the development of cancer in a specific tumor niche.

## MATERIALS AND METHODS

### Materials

IGF-1 and IGFBP3 were obtained from PeproTech Asia (USA). Anti-IGF1R β and anti-phospho-IGF1R (Tyr1135/1136)/insulin receptor (Tyr1150/1151) were from Cell Signaling Technology, Inc. (Danvers, MA). Anti-insulin Rβ and anti-phospho-insulin Rβ were from Santa Cruz Biotechnology (Santa Cruz, CA). Anti-IGFBP3 was from Abcam (Cambridge, UK). The polypeptide GPETLCGAEL was synthesized by Kelowna International Scientific (Taiwan). Matrix-assisted laser desorption ionization–time of flight mass spectrometry (MALDI–TOF) analysis was carried out in the MASS core facility, NRPGM, National Science Council, Taiwan. CL1-0 cells were a gift from Dr Yang Pang-Chyr of the National Taiwan University (27). NCI-H460, H520, and H661 cells were purchased from the American Type Culture Collection and cultured according to instructions.

### Proteolytic processing of IGF-1 by DPP-IV and fibroblast activation protein (FAP) by MALDI-TOF mass spectromtry

To ensure the purity of DPP-IV and FAP, affinity chromatography, ion exchange chromatography, and/or gel filtration chromatography were used to isolate the pure proteases. DPP-IV was purified from baculovirus-infected insect cells, as described previously (3). Ammonium sulfate (3.8 M) was added to medium containing secreted DPP-IV to 70% saturation. The precipitates were resuspended in 50 mM sodium phosphate buffer containing 500 mM NaCl (pH7.4), and the solution was then passed through a nickel affinity column. The column was washed with 10 and 20 mM imidazole before the sample was eluted with 250 mM imidazole, all in buffer containing 50 mM sodium phosphate (pH7.5). Additional purification was performed with ion exchange and gel filtration chromatography. An FAP expression plasmid for insect cells was constructed as described previously (28). FAP was purified with His Bind Resin (Novagen) and HiTrap Q Sepharose FF (GE Healthcare). Specifically, the culture medium of High Five^TM^ cells expressing FAP was collected and the protein precipitated by the addition of 3.8 M ammonium sulfate to 70% saturation. The pellet was resuspended in a binding buffer of 20 mM Tris-HCl buffer (pH8.0) containing 500 mM NaCl, and then passed through a Ni–Sepharose column. A bed volume of binding buffer with 10 mM and 20 mM imidazole was used to wash the column 5–10 times. The FAP protein was eluted with 250 mM imidazole in binding buffer. The eluate was changed to buffer A (20 mM Tris-HCl, pH8.0) with Amicon YM-30 devices (Millipore) before the sample was loaded onto a HiTrap Q Sepharose FF column. Finally, the FAP protein was eluted in a 0–500 mM NaCl gradient in buffer A. The purity of the protein was determined by SDS–PAGE with Coomassie Blue staining. The protein concentration was determined by the Bradford method, using bovine serum albumin (BSA) as the standard.

DPP-IV or FAP was incubated with IGF-1 or peptide in an assay buffer of 10 mM Tris-HCl (pH 8.0) overnight at 37°C. The reaction was terminated by the addition of 15% acetic acid, and the samples were subjected to high-performance liquid chromatography (HPLC) and/or MALDI–TOF analysis, as described previously (29). T-IGF1 was confirmed with MASS analysis.

### Determination of the kinetic constants of the DPP-IV cleavage of IGF-1 by MALDI-TOF mass spectrometry

The kinetic parameters were determined with a published method (30). In this assay, the initial velocity is given as a function of the enzyme concentration [E] with the following equation:

$$\text{V}={\text{k}}_{\text{cat}}{\left[\text{S}\right]}_{0}/(1+{\text{k}}_{\text{m}}{\left[\text{E}\right]}^{-1})$$

where the substrate concentration was kept constant and the enzyme concentration was varied while a large excess of substrate was maintained. Different concentrations of purified DPP-IV (0.4, 0.6, 0.8, 1.2, 1.6, 2.4, 3.6, 4.8, and 6.4 μM) were incubated with 50 nM IGF-1 in an assay buffer of 10 mM Tris-HCl (pH8.0) at a final volume of 100 μL at 37°C over a time course of 5, 10, 20, 30, 40, 50, and 60 min. At the end of the incubation period, the reaction was stopped with acetic acid (15% of the total volume) and the samples were subjected to MALDI–TOF analysis. The peak area was quantified with flexAnalysis (Bruker Daltonik GmbH, Bremen, Germany). The amount of T-IGF1 was calculated by measuring the peak area and comparing it against the IGF-1 control. The initial velocity was calculated from the amount of T-IGF1 obtained at different time points. The kinetic parameters were analyzed using the new Michaelis–Menten equation shown above.

### Cell culture and western blot analysis

CL1-0, NCI-H460, NCI-H520, and NCI-H661 cells were serum starved overnight. Fresh serum-free medium was added and the cells incubated for 1 h before the addition of different concentrations of IGF-1 or T-IGF1. After their incubation with IGF-1 or T-IGF1, the cells were lysed with RIPA buffer (150 mM NaCl, 20 mM Tris, 0.1% SDS, 1% Triton X-100, 1% deoxycholate, 5 mM EDTA, pH7.2) containing protease inhibitor cocktail (Roche) and phosphatase inhibitor cocktail (Sigma). The lysates were clarified by centrifugation, quantified with the Bradford assay (31), mixed and boiled with loading buffer (50 mM Tris-HCl [pH6.8], 0.1% bromophenol blue, 10% glycerol), and fractionated on SDS (10%) polyacrylamide gel. The proteins were transferred to polyvinylidene difluoride membrane by western blotting, and probed with antibody as indicated. The images were analyzed and quantified with Adobe Photoshop using the method outlined at  http://www.lukemiller.org/journal/2007/08/quantifying-western-blots-without.html.

### Relative binding of IGF-1 and T-IGF1 to IGFBP3

Enzyme-linked immunosorbent assays (ELISAs) were used as described previously to determine the relative binding capacities of IGF-1 and T-IGF1 to IGFBP3 (32). A 96-well ELISA plate was coated overnight with 100 μL of either 50 nM T-IGF1 or 50 nM intact IGF-1. FAP protein was mixed with the intact form of IGF-1 and used as the control. Excessive antigen was removed after the overnight incubation, and the nonspecific binding sites were blocked by incubation with 1% BSA in PBS at room temperature for 1 h, followed by three washes with PBS. Then 50, 100, or 200 ng of IGFBP3 in 100 μL of 50 mM Tris-HCl (pH6.8) containing 0.5% BSA was added to the wells. After incubation at 22°C for 2 h with gentle shaking, the unbound IGFBP3 was removed by washing the samples three times with PBS. The plate was incubated for 1 h with 100 μL of an anti-IGFBP3 antibody diluted 1:8000 in PBS, and then washed three times with PBS. A horseradish-peroxidase-conjugated anti-mouse IgG (100 μL), diluted 1:10,000, was added to the wells, and the plate was incubated for another 1 h. After the wells had been washed three times with PBS, the plate was treated with tetramethylbenzidine substrate and the absorbance was determined spectrophotometrically at 620 nm. The relative binding capacity is expressed as a percentage of the absorbance at 620 nM for both intact and truncated IGF-1 relative to that of the control, with different concentrations of IGFBP3. The statistical analysis was performed with Student’s *t* test.

### Quantitative RT–PCR analysis of human lung tissues

Quantitative RT–PCR was performed according to published protocols (33). Tumor and the adjacent nonneoplastic tissues dissected from the lungs of patients with SCC or adenocarcinoma (ADC) were obtained from Chang-Gung Memorial Hospital, Taiwan, according to the institutional guidelines. RNA was converted to cDNA with ImProm-II^TM^ Reverse Transcriptase (Promega) and quantitative PCR was performed with the ABI Prism 7900 Sequence Detection System using the SYBR Green PCR Master Mix (ABI). The sequences of the primers used for the quantitative RT–PCR were: 5′-taaaggaatgccaggaggaa-3′ and 5′-tatagaggggcagaccagga-3′ for DPP-IV, and 5′-gaaggtgaaggtcggagtca-3′ and 5′-tggaagatggtgatgggatt-3′ for GAPDH. The cycle threshold (Ct) of DPP-IV was calculated against a normalization constant derived after correction against GAPDH, as described previously (34). The statistical analysis was performed with Student’s *t* test.

## RESULTS

### Proteolytic cleavage of IGF-1 by DPP-IV

**Figure 1:**
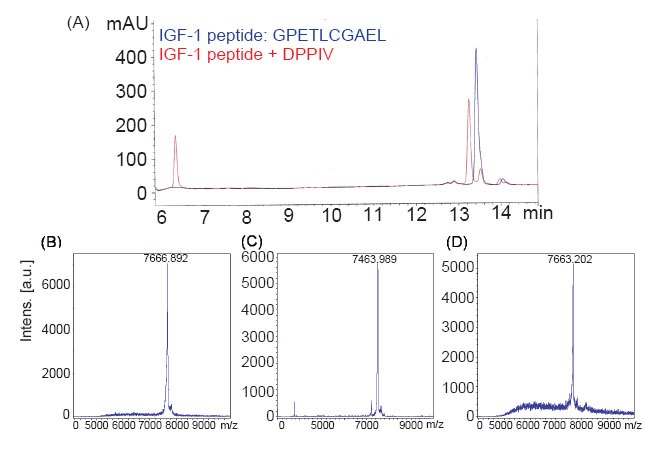
IGF-1 is cleaved by DPP-IV. Panel A: An IGF-1 peptide (10-mer) corresponding to the 10 amino-terminal residues was cleaved by DPP-IV, as shown by HPLC analysis. The original 10-mer is shown in blue, and its cleavage products in red. Panels B–D show a MALDI-TOF analysis of the full-length IGF-1 cleaved by DPP-IV or FAP after incubation for 16 h. Panel B: Full-length IGF-1 only. Panel C: Full-length IGF-1 incubated with DPP-IV. Panel D: Full-length IGF-1 incubated with FAP.

To determine whether DPP-IV cleaves IGF-1, extra precaution was taken to ensure the purity of the proteins used in the study (Materials and Methods). The purified proteins had activities similar to those reported previously (2, 3). FAP is a protease highly homologous to DPP-IV, with 50% sequence identity and structural similarity (28). Therefore, the cleavage of IGF-1 by FAP was also investigated. First, the peptide GPETLCGAEL (the first 10 amino acids at the N-terminus of IGF-1) was incubated with DPP-IV. HPLC analysis identified the dipeptide Gly–Pro and the octapeptide ETLCGAEL as the products of DPP-IV digestion (Fig. 1A). The identities of these two cleavage products were confirmed by MALDI-TOF mass spectrometry (data not shown). Under the same reaction conditions, FAP did not cleave GPETLCGAEL (data not shown). We next investigated whether DPP-IV cleaves full-length IGF-1. IGF-1 has a molecular mass of 7650 daltons (Fig. 1B). After incubation with DPP-IV, the molecular mass of IGF-1 decreased to 7500 daltons, which corresponds to the deletion of the Gly–Pro dipeptide at the amino terminus (Fig. 1C). The molecular mass of IGF-1 did not change when incubated with FAP (Fig. 1D). These data indicate that DPP-IV specifically cleaves the N-terminus of IGF-1, producing the Gly–Pro dipeptide.

### Kinetic constants for the DPP-IV cleavage of IGF-1

The kinetic parameters for the cleavage of IGF-1 by DPP-IV were measured. Because the digestion of IGF-1 by DPP-IV is slow, a specific protocol was used to quantify the enzymatic reaction (30). The initial velocity was measured in the linear range of the reaction. After MASS identification and quantification (Materials and Methods), the *k_cat_/K_m_* value for the cleavage of IGF-1 by DPP-IV was 360 M^–1^s^–1^, with *K_m_* and *k_cat_* equal to 14 μM and 0.005 s^–1^, respectively. Based on these kinetic parameters, this cleavage is more efficient than the cleavage of CXCL11 by matrix metalloproteinases (MMPs) or CD23 by ADAM10, as reported previously (35, 36). At the micromolar level, the *K_m_* value is similar to those for the cleavage of GLP1 and GIP by DPP-IV (5, 7, 8). Therefore, the cleavage of IGF-1 by DPP-IV is physiologically relevant, despite its slow reaction, indicated by the *k_cat_* value.

### Reduced and delayed activation of IGF-1R by T-IGF1

**Figure 2:**
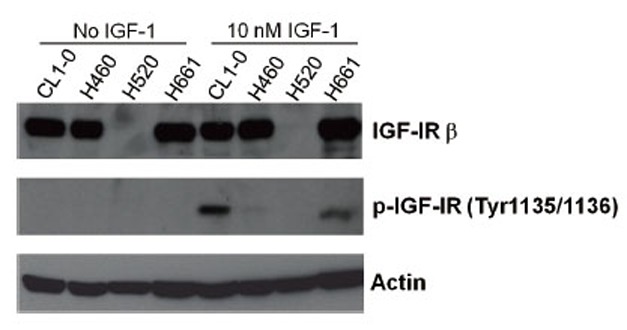
Activation of IGF-1R by IGF-1 in different lung cancer cell lines. (A) Serum-starved CL1-0, NCI-H460, NCI-H520, and NCI-H661 cells were stimulated with IGF-1 for 30 min and the phosphorylation status of IGF-1R was determined by western blot analysis. The concentration of IGF-1 is indicated on the top of each lane. The antibodies used in the western blot analysis are indicated on the right side of the panel.

The signal transduction of the IGF signaling pathway is initiated by the activation of IGF-1R by IGF-1. To study the interaction between IGF-1R and intact IGF-1 or T-IGF1, the activation of IGF-1R by IGF-1 was first investigated in different human lung cancer cell lines, including CL1-0 (a well-studied lung ADC cell line (27)), NCI-H520 (a lung SCC cell line), NCI-H460 and NCI-H661 cells (two large-cell carcinoma cell lines). IGF-1R is expressed abundantly in all the cell lines studied, except NCI-H520. Interestingly, the activation of IGF-1R was only detected in the CL1-0 and NCI-H661 cells in the presence of 10 nM IGF-1 after stimulation for 30 min (Fig. 2). Very weak activation of IGF-1R was detected in the NCI-H460 cells (Fig. 2). Therefore, CL1-0 cells were selected to study the activation of IGF-1R by intact or truncated IGF-1.

**Figure 3:**
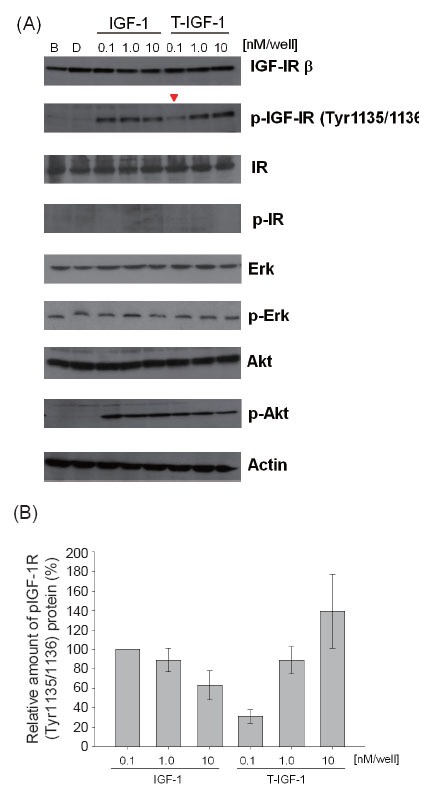
Reduced activation of IGF-1R by T-IGF1. (A) Serum-starved CL1-0 cells were stimulated with the indicated concentrations of IGF-1 or T-IGF1 for 5 min and the phosphorylation status of IGF-1R, IR, ERK, AKT, and actin was determined by western blot analysis. The concentration of IGF-1 or T-IGF1 is indicated on the top of each lane. B: Blank control. D: DPP-IV only added. The antibodies used in the western blot analysis are indicated on the right side of the panel. (B) Quantitation of the phospho-IGF-1R (Tyr1135/1136) protein levels during treatment with different concentrations of either IGF-1 or T-IGF1. For each lane, the phospho-IGF-1R (Tyr1135/1136) level was normalized to that of actin. The results are an average of three experiments, with error bars representing the standard deviations.

IGF-1 at concentrations of 0.1–10 nM increased the phosphorylation of IGF-1R at Tyr 1135/1136 in CL1-0 cells compared with that in untreated controls (lane B, Fig. 3A) or cells treated with purified DPP-IV only (lane D, Fig. 3A). As shown in Fig. 3B, this phosphorylation was significantly reduced in cells treated with 0.1 nM T-IGF1 (0.1 nM, a physiological concentration) (23, 26). There was no significant difference between IGF-1 and T-IGF1 in activating IGF-1R when their concentrations were higher than the physiological levels (0.5 nM and 1 nM; Fig. 3A). These results indicate that T-IGF1 is less potent than intact IGF-1 in activating IGF-1R. There was no difference in the phosphorylation of downstream AKT or ERK when either IGF-1 or T-IGF1 was added at any concentration tested (Fig. 3).

**Figure 4:**
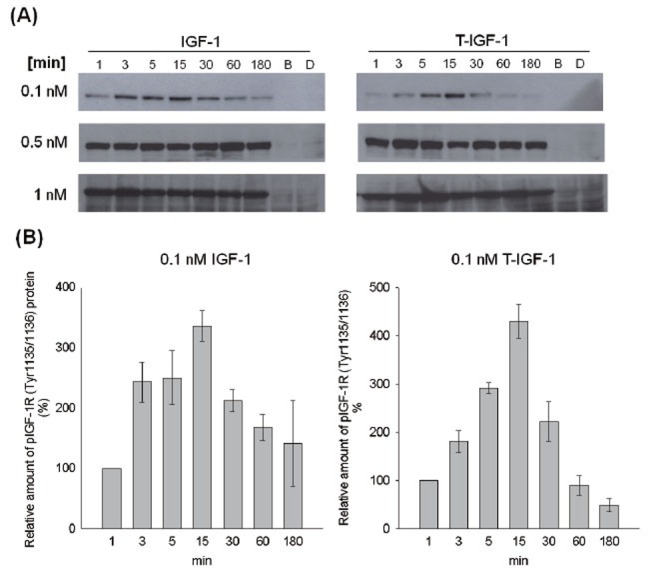
Delayed and less-sustained activation of IGF-1R by T-IGF1. Serum-starved CL1-0 cells were stimulated with the indicated concentrations of IGF-1 or T-IGF1 for different periods (from 0 to 180 min), and the phosphorylation status of IGF-1R was determined by western blot analysis. The concentrations of IGF-1 and T-IGF1 are indicated on the left side of the panels. B: Blank control. D: DPP-IV only added. Panel A: Activation of IGF-1R by IGF-1 (left) or T-IGF1 (right). Panel B: Quantitation of phospho-IGF-1R (Tyr1135/1136) protein levels during treatment with 0.1 nM IGF-1 (left) or T-IGF1 (right). For each lane, the phospho-IGF1R (Tyr1135/1136) level was normalized to that of actin. The results are the averages of three experiments, with error bars representing the standard deviations.

The temporal response of IGF-1R to IGF-1 or T-IGF1 was determined (Fig. 4A). For intact IGF-1 at 0.1 nM, IGF-1R phosphorylation reached an optimal level 3 min after stimulation, was sustained for 15 min, and decreased after 30 min (Fig. 4). Three hours after stimulation, increased IGF-1R phosphorylation remained detectable (Fig. 4). However, for T-IGF1 at a similar concentration, the peak level of activation was not reached for 15 min and was followed by a large drop at 30 min (Fig. 4). No IGF-1R activation was observed after 3 h (Fig. 4). When IGF-1 or T-IGF1 was added at concentrations higher than physiological levels (0.5 and 1 nM), the levels of IGF-1R phosphorylation caused by both were indistinguishable during the subsequent 3 h. The results shown in Fig. 4 suggest that the activation of IGF-1R by T-IGF1 is delayed and not sustained.

### Increased binding between T-IGF1 and IGFBP3

**Figure 5:**
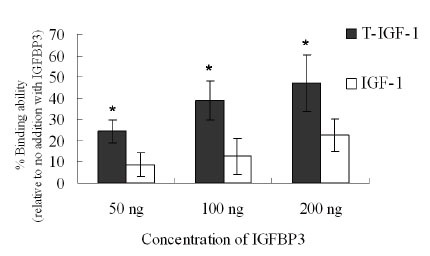
Stronger binding of T-IGF1 to IGFBP3. The binding of IGFBP3 at different concentrations to either T-IGF1 or IGF-1 was measured by ELISA, as described in the Materials and Methods. The values are the means ± standard errors of three independent experiments. *P<0.005.

Because IGFBP3 is the major protein sequestering IGF-1, the relative binding affinities of IGF-1 and T-IGF1 for IGFBP3 were determined by ELISA. As shown in Fig. 5, the amount of T-IGF1 binding to IGFBP3 was 2–3 times greater than the amount of IGF-1 binding to IGFBP3. These data indicate that the binding affinity of T-IGF1 for IGFBP3 is stronger than that of IGF-1, and therefore IGFBP3 sequesters T-IGF1 more effectively than IGF-1 from the cognate IGF-1R.

### Downregulation of DPP-IV in human SCC but not ADC

**Figure 6:**
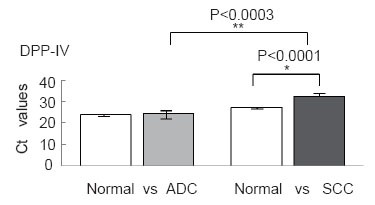
Expression of DPP-IV in ADC and SCC patients measured by quantitative RT–PCR. Ct values with standard deviations are given. Differences in gene expression levels between normal tissue and ADC and between normal tissue and SCC were analyzed with Student’s *t* test. *P<0.0001, **P<0.0003.

Given that IGF-1 truncation by DPP-IV affects the activation of IGF-1R, we next investigated the expression levels of DPP-IV in human non-small-cell lung cancer tissues with quantitative RT–PCR. In each of six patients with lung ADC and six patients with lung SCC, one specimen was taken from the cancerous tissue and another from the adjacent nonneoplastic tissue. Quantitative RT–PCR showed a dramatic reduction in DPP-IV, to an almost undetectable level, in all six SCC samples (Fig. 6). In comparison, no statistically significant variation in DPP-IV levels was seen in any of the six ADC samples (Fig. 6).

## DISCUSSION

**Figure 7:**
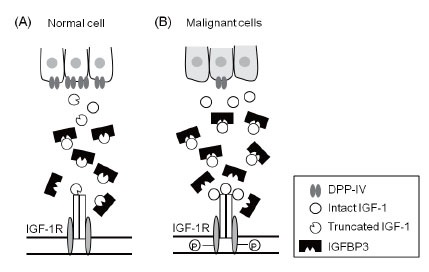
Model of DPP-IV, IGF axis, and cancer formation. Panel A: In normal cells, DPP-IV mitigates IGF1 activity by cleaving IGF-1 to T-IGF1, resulting in the delayed and less-sustained activation of IGF-1R and the greater sequestration of T-IGF1 by IGFBP3, as suggested by the data presented in this paper. Panel B: In malignant cells, the downregulation of DPP-IV might result in elevated levels of intact IGF-1 in the tumor niche, activating the IGF-1 axis. This might contribute to the acceleration of early carcinogenesis in certain cancer subtypes, such as SCC, as presented in this study.

The cancer microenvironment is fundamentally different from that of normal tissues. The prevailing model is that activated fibroblasts in tumors modify the phenotype of the epithelial cells via soluble and diffusible growth factors (37). These growth factors, including IGF-1, accelerate cell proliferation, modify the extracellular matrix, promote angiogenesis, and increase inflammatory cell recruitment (37). The proteolytic processing of IGF-1 by DPP-IV may represent a defense mechanism that mitigates IGF-1 activity in normal cells (Fig. 7A). Here, we provide evidence that IGF-1 activity is regulated by proteolytic processing by DPP-IV. The attenuation of IGF-1 signaling may be achieved by both a reduction in IGF-1R activation and increased IGF-1–IGFBP3 binding (Figs 3–5). The *K_m_* of IGF-1 cleavage by DPP-IV is similar to the range reported for other physiological substrates of DPP-IV, including GLP1 and GIP (5–8). Consequently, IGF-1 could be a biological substrate of DPP-IV. Finally, DPP-IV is downregulated to almost undetectable levels in SCC, whereas there is no significant change in DPP-IV levels in ADC, suggesting that separate mechanisms underlie the development of these two cancer subtypes.

This dramatic reduction of DPP-IV in SCC suggests that an elevated level of intact IGF-1 may be present in the tumor niche of this cancer subtype (Fig. 7B). IGF-1 plays a critical role in the acceleration of early carcinogenesis (19). The overexpression of IGF-1 is associated with hyperplasia and neoplasia at an early stage in several animal models. On the contrary, reduced IGF-1 expression is linked to a reduction in cancer formation and metastasis (38, 39). In human population studies, higher IGF-1 levels in the circulation are associated with some cancer types, including colorectal, prostate, and breast cancers (40–42). Because the expression levels and regulation of IGF-1 affect cancer formation and progression (19), IGF-1 homeostasis in the microenvironment is crucial to the maintenance of the proper cellular proliferative and inhibitory activity. Higher levels of intact IGF-1 in the absence of DPP-IV might contribute to the early carcinogenesis of lung SCC.

The catalytic efficiency of DPP-IV for IGF-1 is close to the reported catalytic efficiencies of MMP and ADAM10 (*k_cat_/K_m_* = 360 M^–1^s^–1^ for DPP-IV against IGF-1; 100–200 M^–1^s^–1^ for MMPs against CXCL11 (43); and 90 M^–1^s^–1^ for ADAM10 against CD23 (36)). Such slow catalytic rates might be characteristic of and critical for these extracellular proteases to maintain the homeostasis of the stromal microenvironment. The cleavages are slow and take hours. It is unclear whether this is the reason for the past failure to observe IGF-1 cleavage by DPP-IV *in vitro* (7). Recently, Faidley *et al*. (2006) reported no significant change in circulating IGF-1 levels when the DPP-IV inhibitor sitagliptin was administered to pigs for three days (44). It remains to be investigated how stable T-IGF1 is *in vivo* and whether these measurements are the composite of both intact and truncated IGF-1 proteins.

The interactions between T-IGF1 and IGFBP3/IGF-1R, as shown in our study, are also consistent with the results of crystallization and various biochemical studies (45–47). The residues that interact with IGFBPs are located at the B (residues 1–29) and A domains (residues 42–62) of IGF-1 (45). Based on the cocrystal structure of IGF-1 with either the N-terminal or C-terminal domain of IGFBP, several residues of IGF-1 seem to be engaged in the interaction (46). Mutations at these residues, such as Glu3, significantly reduce the binding of IGF-1 to IGFBPs (48). Site-directed mutagenesis and binding assays have suggested that the binding of IGF-1 to IGF1R occurs between the C and D domains of IGF-1, which differs from the binding between IGF-1 and IGFBPs (46). Therefore, the weaker activation of IGF-1R by T-IGF1 is probably attributable to the conformational change caused by the lack of two amino acids at the amino terminus.

Reduction of the ligand concentration and a blockade of IGF1–IGF1R binding are promising anticancer strategies (26, 49). Our results suggest that the proteolytic processing of IGF-1 is another alternative way to control the IGF axis. Consistent with this, the expression of the DPP-IV protease is reduced in certain cancer types (e.g., human lung SCC). Because DPP-IV inhibitors are currently used to treat human type II diabetes (50, 51), their potential long-term impact on the balance between cell growth and apoptosis in this niche warrants further investigation.
